# A darker chromatic variation of *Rhodnius pallescens* infected by specific genetic groups of *Trypanosoma rangeli* and *Trypanosoma cruzi* from Panama

**DOI:** 10.1186/s13071-018-3004-4

**Published:** 2018-07-16

**Authors:** Azael Saldaña, Ana María Santamaría, Vanessa Pineda, Vanessa Vásquez, Nicole L. Gottdenker, José E. Calzada

**Affiliations:** 1 Instituto Conmemorativo Gorgas de Estudios de la Salud (ICGES), Ciudad de Panamá, Panamá; 2 Centro de Investigación y Diagnóstico de Enfermedades Parasitarias (CIDEP), Facultad de Medicina, Universidad de Panamá, Ciudad de Panama, Panamá; 3 Department of Veterinary Pathology, University of Georgia College of Veterinary Medicine, Athens, Georgia 30602 USA

**Keywords:** *Rhodnius pallescens*, *Trypanosoma cruzi*, *Trypanosoma rangeli*, Genotyping, Panama

## Abstract

**Background:**

*Rhodnius pallescens*, the only species of this genus reported in Panama, has a wide geographical distribution and is associated with most cases of Chagas disease and human infections with *Trypanosoma rangeli* in this country. Thus far, no phenotypic variants of this triatomine have been registered. Similarly, genotyping of the trypanosomes that infect this vector has only been partially evaluated.

**Results:**

A total of 347 specimens of *R. pallescens* were collected in *Attalea butyracea* palm trees located near a mountainous community of the district of Santa Fe, province of Veraguas. Bugs were slightly longer and had a darker coloration compared to that reported for this species. Infection rates for trypanosomes performed with three PCR analyses showed that 41.3% of the adult triatomines were positive for *T. cruzi*, 52.4% were positive for *T. rangeli* and 28.6% had mixed *T. cruzi*/*T. rangeli* infections. Based on *cox*2 analysis, TcI was the single *T. cruzi* discrete typing unit (DTU) detected, and a genetic variant of KP1(-)/lineage C was the only genetic group found for *T. rangeli*.

**Conclusions:**

A darker chromatic variation of *R. pallescens* predominates in a mountainous region of Panama. These triatomines show high trypanosome infection rates, especially with *T. rangeli.* Regarding *T. rangeli* genetic diversity, complementary studies using other molecular markers are necessary to better define its phylogenetic position.

## Background

Human infection with *Trypanosoma cruzi* and/or *Trypanosoma rangeli* has been reported in different regions of the Republic of Panama [1]. *Trypanosoma cruzi* is the causal agent of Chagas’ disease, a zoonosis most commonly associated in Panama with heart sickness [2]. *Trypanosoma rangeli* is a parasite not linked to pathological processes in humans [3], but with clinical and epidemiological importance due primarily to its antigenic similarity with *T. cruzi* [4]. Both trypanosomes are transmitted by triatomine bugs (Reduvidae: Triatominae) of wide distribution in different habitats throughout the country [5]. *Rhodnius pallescens* is the main vector of *T. cruzi* and *T. rangeli* in Panama [1]. The principal biotope for *R. pallescens* is the crown of the “royal palm” (*Attalea butyracea*), from where it frequently moves to nearby dwellings.

Intraspecific genetic variability has been described in both *T. cruzi* and *T. rangeli* isolates using several molecular markers [3, 6]. Genotyping of these hemoparasites, as well as the study of the biological variants adopted by their vectors in an endemic region, is important to achieve the understanding, surveillance and management of particular eco-epidemiological scenarios of Chagas’ disease. In this report, we describe phenotypic characteristics of a darker chromatic population of *R. pallescens*, infection rates, and trypanosome genotypes that infect this melanic variant.

## Methods

The study took place in a rural Panamanian community called La Culaca (8°31'0"N, 81°2'60"W, 698 masl), located in the Northern region of the Veraguas Province, Santa Fe District, in the western half of the Isthmus of Panama. This region was recently described as a new focus of Chagas disease in Panama [7]. Triatomine bugs were captured during dissection of nine *A. butyracea* palm trees located less than 100 m from inhabited community dwellings. Palms were cut down by their owners for agricultural activities, collection of leaves for roofing, and/or preparation of “palm wine”. The number of bugs was counted from each palm, and developmental stage, species and morphological characteristics such as length and coloring patterns were described for each individual triatomine collected.

The DNA of the adult triatomines was extracted from the whole vector according to a methodology previously described that has proven to be efficient for further molecular analysis [8]. To evaluate natural trypanosome infections, two independent PCR analyses based on genes encoding for H2A/SIRE (TcH2AF/R) and sno-RNA-C11 (TrF/R2) were carried out [9]. The primers TcH2AF/R and TrF/R2 amplify 234 bp and 620 bp fragments for *T. cruzi* and *T. rangeli*, respectively. In addition, a restriction fragment length polymorphism (RFLP) analysis based on the cytochrome *c* oxidase subunit 2 gene (PCR/RFLP-*cox*2) was used for the simultaneous detection and typing of *T. rangeli* major genetic groups and *T. cruzi* discrete typing units (DTUs) [10]. PCR-*cox*2 products from 8 *T. rangeli* and 9 *T. cruzi* single infections were further purified and sequenced using the same primers. Sequences were edited and compared with retrieved reference sequences representing each *T. rangeli* major genetic group and *T. cruzi* DTUs in GenBank by Basic Local Alignment Search Tool (BLAST) queries.

## Results and discussion

All palms were infested with *R. pallescens*. In total, 347 specimens were collected, including 63 adults and 284 nymphs (16 fifth-instar, 90 fourth-instar, 113 third-instar, 52 second-instar and 13 first-instar). All fifth-, fourth- and third-stage nymphs were examined by microscopy for trypanosome infection. We did not test first- and second-stage nymphs due to the technical difficulty to process individually these specimens for microscopic examination.

**Fig. 1:**
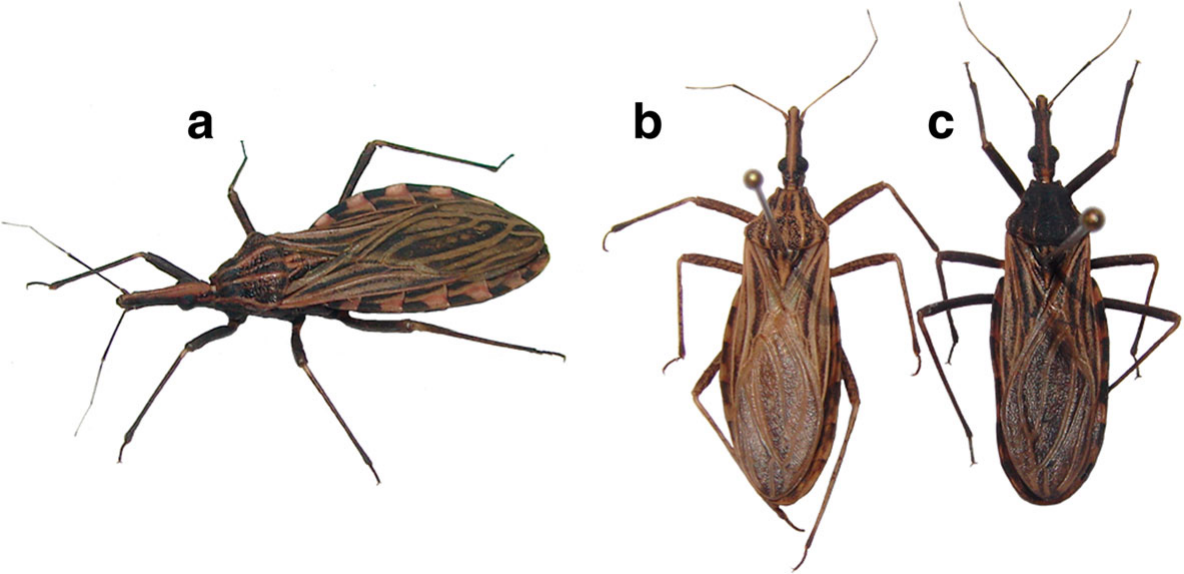
Chromatic variations of *Rhodnius pallescens* in Panama. **a** Common darker specimen from Santa Fe, Veraguas Province, Panamá. **b** Specimen with a typical coloration found in other Chagas disease endemic regions. **c** Completely dark specimen from Santa Fe, Panamá

Of the 63 adults, 38 were males (60.3%) and 25 females (39.7%). Adult bugs had a darker coloration and an average length slightly longer (22–24 mm for males and 22.5–25 mm for females) than the standard established for this species [11] (Fig. 1). Rectangular spots of dorsal connexival plates, an important morphological characteristic of *R. pallescens* [11], were clearly distinguishable. However, the legs of both adults and nymphs were uniformly blackish. The mottled pattern typically described for *R. pallescens* legs was not observed (Fig. 1). Three (4.8%) of the adult bugs collected were completely dark, with a pronotum entirely black and the abdomen with an under surface of a general dark brown color (Fig. 1).

**Fig. 2:**
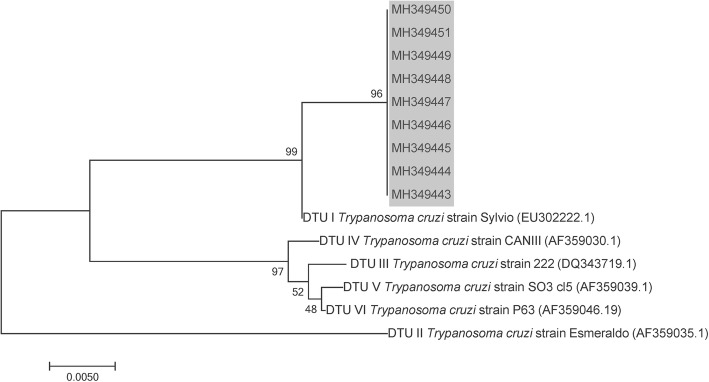
Phylogenetic positioning of *Trypanosoma cruzi* parasites found in a darker chromatic variation of *Rhodnius pallescens* from Santa Fe, Veraguas Province, Panamá. The phylogenetic tree is based on cytochrome *c* oxidase subunit 2 sequences. The trees were constructed using the neighbor-joining method and numbers on the tree indicate bootstrap values for branch points. The analyses included reference sequences of *T. cruzi* discrete typing units (DTUs) (accession numbers in parentheses). The gray shaded portion of the tree corresponds to the sequences generated in this study

**Fig. 3:**
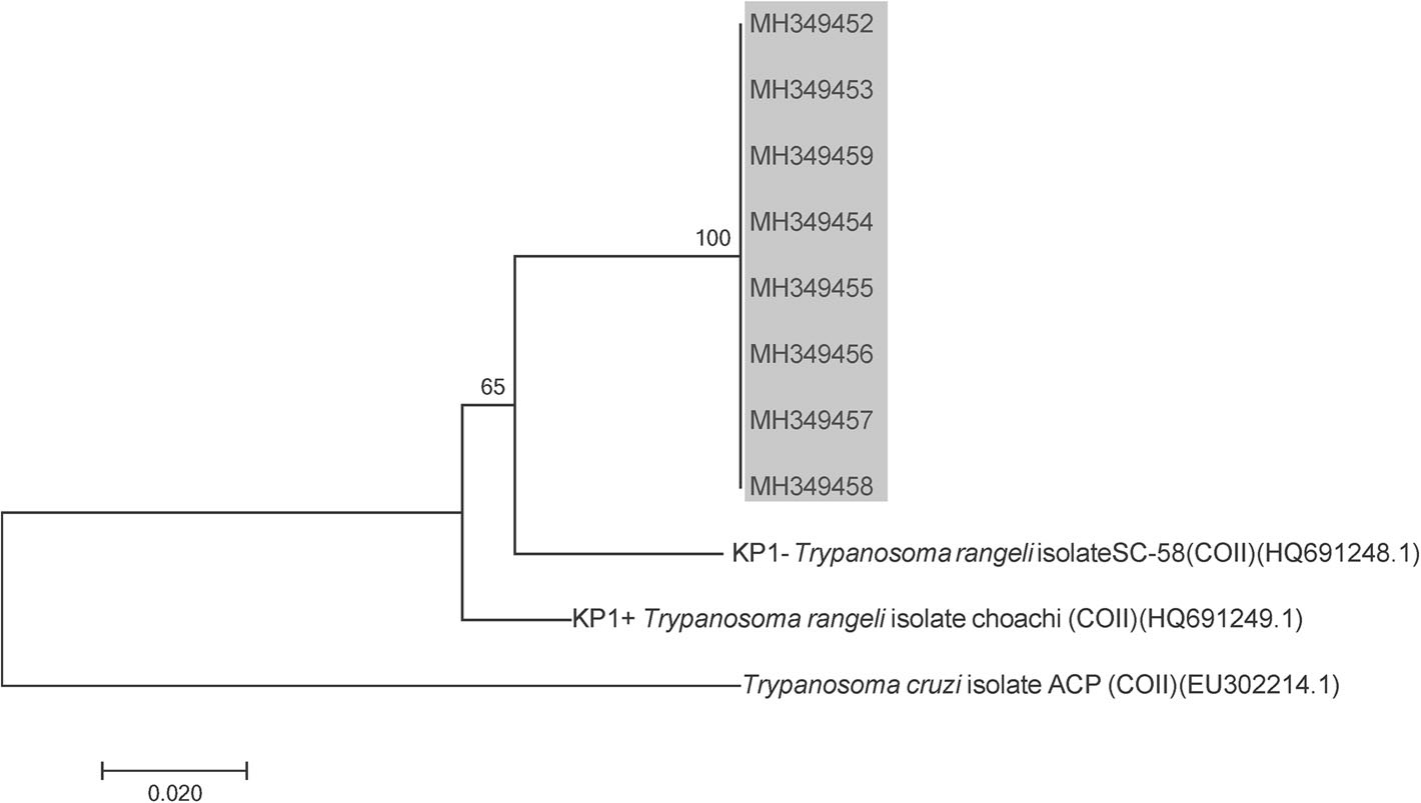
Phylogenetic positioning of *Trypanosoma rangeli* parasites found in a darker chromatic variation of *Rhodnius pallescens* from Santa Fe, Veraguas Province, Panamá. The phylogenetic tree is based on cytochrome *c* oxidase subunit 2 sequences. The trees were constructed using the neighbor-joining method and numbers on the tree indicate bootstrap values for branch points. The analyses included reference sequences of *T. rangeli* major genetic groups (accession numbers in parentheses). The gray shaded portion of the tree corresponds to the sequences generated in this study

Infection rates for trypanosomes detected by microscopy in fifth-, fourth- and third- instar nymphs were 18.8%, 58.9% and 64%, respectively. Due in part to economic constraints, we focused our molecular analysis in adult specimens, which for this particular sylvatic species have a major vector relevance in the domestic transmission scenario. In adult bugs, infection rates for trypanosomes were performed using three PCR analyses (TcH2AF/R, TrF/R2 and PCR/RFLP-*cox*2). The combined results showed that 41.3% (26/63) of the adult triatomines were positive for *T. cruzi*, 52.4% (33/63) were positive for *T. rangeli* and 28.6% (18/63) had mixed *T. cruzi*/*T. rangeli* infection. The rate of single infections was lower; 14.3% (9/63) were positive for *T. cruzi* only and 22.2% (14/63) were singly infected with *T. rangeli*. Twenty-two (34.9%) triatomines did not have trypanosome infections. *Trypanosoma rangeli* and *T. cruzi* infections were genotyped by PCR/RFLP-*cox*2 and confirmed by sequencing PCR-*cox*2 amplicons. *Trypanosoma cruzi* lineage I (TcI) was the single *T. cruzi* discrete typing unit (DTU) detected, and KP1(-)/lineage C was the only genetic group found for *T. rangeli* (Figs. 2, 3). Interestingly, the phylogenetic relationships assessed by *cox*2 partial sequencing confirmed that *T. rangeli* isolates from this Panamanian region were more closely related to KP1(-); however, they all grouped in a separate clade independently of the KP1(-) reference strain (Fig. 3).

So far, 21 species of *Rhodnius* have been described [13], almost all of them are associated with palm tree habitats. *Rhodnius pallescens* infestation rates in *A. butyracea* palms from Panama are the highest reported among triatomines infesting palms throughout Latin America [12]. This was also demonstrated in the search conducted during the present study, where 100% of the palms evaluated were infested with this triatomine. Even though the chromatic patterns are useful for *Rhodnius* species identification [11], some interspecific chromatic variations have been reported for this species [14–16], as in other triatomine groups [17–19]. However, as far as we know, there were no reports of chromatic differences in *R. pallescens*. In this species, the typical specimens show a general yellowish brown color with dark brown spots, in many cases dotted lighter [11] (Fig. 1). The occurrence of a darker chromatic variation of *R. pallescens* observed in the mountainous region of Santa Fe, Panama (Fig. 1) could be an example of how the biotope inhabited by this species can influence its coloration. This melanic bugs blends perfectly with the wet crowns of *A. butyracea*, common of this rainy region with cooler temperatures compared to other areas where *R. pallescens* has been reported in Panama. This adaptive character (camouflage) for a darker habitat could be very useful to avoid natural predators present in these palms. The larger average size of these bugs could also be considered a particular adaptation to this ecological niche. In this regard, the difference in coloration has been considered as an important trait for the recent descriptions of two new *Rhodnius* species [13, 20]. Nevertheless, additional genetic, morphometric, cross-breeding and geographical distribution studies are needed to clarify whether the darker variant of *R. pallescens* from Santa Fe represents a different phenotypic variation of the typical specimens or a distinct species. It is also important to mention that no typical specimens of *R. pallescens* have yet been found in Santa Fe region.

Another objective of this study was to determine the infection rates and genetic diversity of the trypanosome populations that are circulating in the darker variation of *R. pallescens* described above. The rate of infection with *T. rangeli* and/or *T. cruzi* was high (65.1%) in the dark specimens, similar to other investigations from Panama that evaluated infection index with trypanosomes in *R. pallescens* [8, 21, 22]. It is interesting to note that the infection rate with *T. cruzi* was lower (41.3%) than that reported for *R. pallescens* collected from others endemic areas in Panama [8, 22]. However, the rate found for the infection with *T. rangeli* is one of the highest reported in Panama and other regions where this parasite has been described [9, 23–25]. These high infection frequencies with *T. rangeli* seem to be inconsistent with the described pathological effects of *T. rangeli* for its vectors [26]. Recently, Peterson & Graham [27] also called attention to the lack of sufficient evidence to support the assumption that *T. rangeli* is pathogenic to all *Rhodnius* species. On the other hand, Gottdenker et al. [28] postulated that *T. rangeli*/*T. cruzi* co-infection may confer a survival advantage for *R. pallescens* throughout adulthood. The same conclusion was reached by Peterson & Graham [27] who observed that *R. prolixus* experimentally co-infected with *T. cruzi* and *T. rangeli* have higher survival than bugs infected with just one of the species. In any case, a better evaluation of the occurrences and consequences of the infection with *T. cruzi* and/or *T. rangeli* in the dark variant of *R. pallescens* is necessary. Regarding trypanosome genetic diversity, TcI was the only DTU detected for *T. cruzi* and Kp1(-)/lineage C for *T. rangeli* (Fig. 2, 3). Both results concur with previously conducted studies on the genetic characterization of human trypanosomes in Panama [29–31]. In other Latin American endemic regions, TcI is the most frequently *T. cruzi* DTU in sylvatic cycles, but it is also found in domestic epidemiological scenarios [32]. On the other hand, this study describes the presence of *T. rangeli* Kp1 (-)/lineage C in *R. pallescens* from Panama, an expected result considering the geographical distribution and the association with the “pallescens group” described for this genotype [33]. However, phylogenetic analyzes show some differences between the *T. rangeli* evaluated and the *cox*2 sequences so far described for *T. rangeli* Kp1(-) isolates (Fig. 3). In this regard, it is important to consider the reported association between genetic groups of *T. rangeli* and some *Rhodnius* species conditioned to specific geographical regions and different ecotopes [34]. In this sense, subtyping these Tc1 and Kp1(-) parasites [35, 36] must also be considered for a better knowledge of the intraspecific genetic variability adopted by the trypanosomes that infect *R. pallescens* in the mountainous region of Santa Fe, Panama.

## Conclusions

In this report we provide information on basic phenotypic characteristics, trypanosome infection and trypanosome genotypes infecting a darker chromatic population of *R. pallescens* found in a recently described area that is endemic for Chagas disease in Panama. These adult triatomines showed high trypanosome infection rates, particularly with *T. rangeli.* A better evaluation of the consequences of *T. rangeli* infection in this chromatic variant of *R. pallescens* is needed. The genetic diversity of these trypanosomes was partially studied. TcI was the only DTU detected for *T. cruzi*, and Kp1(-)/lineage C for *T. rangeli*. However, based on *cox*2 sequences analysis, it is suggested that the described *T. rangeli* genetic variant needs to be further characterized using other molecular markers.

## Abbreviations

BLAST: Basic Local Alignment Search Tool
*cox*2: Cytochrome *c* oxidase subunit 2
DTU: Discrete typing unit
RFLP: Restriction fragment length polymorphism
TcI: *Trypanosoma cruzi* lineage I
